# Analysis of Crossover Events and Allele Segregation Distortion in Interspecific Citrus Hybrids by Single Pollen Genotyping

**DOI:** 10.3389/fpls.2020.00615

**Published:** 2020-05-25

**Authors:** Miguel Garavello, José Cuenca, Steven Dreissig, Jörg Fuchs, Luis Navarro, Andreas Houben, Pablo Aleza

**Affiliations:** ^1^Centro de Citricultura y Producción Vegetal, Instituto Valenciano de Investigaciones Agrarias, Valencia, Spain; ^2^Concordia Agricultural Experiment Station, National Agricultural Technology Institute, Entre Ríos, Argentina; ^3^Department of Breeding Research, Leibniz Institute of Plant Genetics and Crop Plant Research (IPK), Seeland, Germany; ^4^Institute of Agricultural and Nutritional Sciences, Faculty of Natural Sciences III, Martin Luther University Halle-Wittenberg, Halle (Saale), Germany

**Keywords:** fluorescence-activated cell sorting, whole genome amplification, SSR and SNP markers, mandarin, lemon

## Abstract

In citrus, a classical method of studying crossovers and segregation distortion (SD) is the genetic analysis of progenies. A new strategy combining fluorescence-activated cell sorting and whole genome amplification of haploid pollen nuclei with a large set of molecular markers, offers the opportunity to efficiently determine the frequency of crossovers and the identification of SD without the need to generate segregating populations. Here we have analyzed meiotic crossover events in a pollen nuclei population from “Eureka” lemon and the allelic SD was evaluated in a pollen nuclei population from a clementine × sweet orange hybrid (“CSO”). Data obtained from the “CSO” pollen nuclei population were compared to those obtained from genotyping of a segregating population (“RTSO”) arising from a hand-made sexual hybridization between diploid non apomictic selected tangor (mandarin × sweet orange; “RTO” tangor) as female parent pollinated with “CSO” tangor as male parent. The analysis of crossovers rates on chromosome 1 revealed the presence of up to five crossovers events on one arm and four on the corresponding other arm, with an average of 1.97 crossovers per chromosome while no crossover events were observed in five “Eureka” lemon pollen nuclei. The rate of SD observed in “CSO” pollen nuclei (13.8%) was slightly lower than that recovered in the “RTSO” population (20.7%). In the pollen nuclei population, SD was found on linkage group (LG) 2, while the “RTSO” population showed SD on LGs 2 and 7. Potential male gametic selection mechanisms were distinguished in pollen grains, while in the population, mechanisms of gametophytic selection and/or zygotic selection were observed. This methodology is a very useful tool to facilitate research focused on the reproductive biology of citrus and study the mechanisms that affect crossovers and SD.

## Introduction

Most plant species that reproduce sexually alternate the life cycle between a diploid sporophytic phase and a reduced gametophytic haploid phase (Xu et al., 2013). Meiosis is the process in which the number of chromosomes is halved, leading to haploid gametes (Zamariola et al., 2014; Bomblies et al., 2015). The formation of crossovers between homologous parental chromosomes occurs in the prophase of meiosis I (Hunter, 2015; Lambing et al., 2017; Li et al., 2017), and consists in the exchange of genetic information between two non-sister homologous chromatids (Zamariola et al., 2014; Lambing et al., 2017). Crossovers are fundamental for chromosome segregation in most eukaryotes, but play also an important role in evolution and speciation of plants (Li et al., 2017), as they generate novel genetic combinations (Morgan et al., 2017). Many studies have demonstrated that the distribution of meiotic crossover events along chromosomes is non-random in plants, varying locally and displaying several hotspots (Mézard, 2006; Choi et al., 2008; Lambing et al., 2017).

On the other hand, allelic segregation distortion (SD), that is, the deviation of the allelic segregation ratio from the expected Mendelian-ratio, is increasingly recognized as an additional powerful evolutionary force (Dai et al., 2017). SD may result mainly from non-random segregation of gametes during meiosis (due to several causes such as the presence of deleterious alleles or gamete competition), post-meiotic gamete dysfunction or differential gamete success (such as differential pollen tube growth, pollen–pistil interactions, or partial incompatibility) and differential zygotic fitness (Li et al., 2014; Bodénès et al., 2016; Fishman and McIntosh, 2019; Seymour et al., 2019).

Many studies have been conducted in crossover formation and allelic SD in several plant species, such as *Arabidopsis* spp., wheat, barley, maize, tomato, and rice (Zamariola et al., 2014; Wang and Copenhaver, 2018). In citrus species, belonging to the family *Rutaceae*, subfamily *Aurantioideae* and with nine (*x* = 9) as basic chromosome number (Krug, 1943), the analysis of the crossover rate was determined in mandarin and lemon by analyzing progenies with molecular markers. Results showed that up to four crossover events per chromosome arm can be found (Cuenca et al., 2011; Aleza et al., 2015; Rouiss et al., 2017b). In addition, skewed markers appear to be concentrated in specific areas, usually named SD regions, which are different for the different genotypes analyzed (Ollitrault et al., 2012a), and could contain deleterious genes or be associated with hybridization incompatibility (Zhou et al., 2015). In addition to parental genotype, differential aptitude of gamete genotypes, direction of the crosses, and regulatory gene interactions can also contribute to the high level of SD in citrus (Bernet et al., 2010; Curtolo et al., 2017). Such SD was proposed to result from gametic selection rather than zygotic selection for the analyzed crosses (Bernet et al., 2010; Ollitrault et al., 2012a).

A classical approach to locate crossovers is analyzing the co-inheritance of linked heterozygous markers through meiosis in segregating populations, which requires precise genetic maps and the availability of known genome structures (Choi and Henderson, 2015). Strategies for identifying whether gametic or zygotic selection (or both) is causing SD are based on genotyping segregating populations from reciprocal and mixed crosses (Ruiz and Asins, 2003; Bernet et al., 2010; Ollitrault et al., 2012a; Xu et al., 2013; Bodénès et al., 2016; Fishman and McIntosh, 2019; Seymour et al., 2019). Alternative methods involve cytogenetic analyses based on recombination nodule maps, chiasma counting or C-band maps (Mézard, 2006; Li et al., 2017).

One alternative strategy for investigating both meiotic recombination patterns and allelic SD is to perform direct genotyping of the gametes, rather than individuals from segregating populations. This methodology paves the way for new information into the genetic basis of non-Mendelian inheritance in contrast to population-based analyses (Choi et al., 2008; Dreissig et al., 2015, 2017; Sun et al., 2019).

The use of the fluorescence activated cell sorting (FACS) technique (Galbraith, 2010) coupled with whole genome amplification (WGA) allows the analysis of individual nuclei with a high number of molecular markers (Dreissig et al., 2015, 2017). In this context, genotyping of individual pollen grains nuclei can be useful for the determination of the male parent haplotypes, the evaluation of meiotic recombination and potential allelic SD (Mase et al., 2014; Dreissig et al., 2017). This strategy also allows performing studies on the genetic structures of pollen grain populations as compared with those originated at the plant level, without interferences due to a potential partial cross-incompatibility or gamete competition (Gu et al., 2013). In citrus, the strategy of ploidy level analysis, sorting, and genotyping of single pollen grains has been previously assessed for several diploid, triploid, and tetraploid genotypes (Garavello et al., 2019), with successful results when applied to haploid pollen grains.

In the current study, we used a strategy combining flow-sorting of pollen nuclei with WGA and genotyping of individualized nuclei by simple sequence repeats (SSR) and single nucleotide polymorphism (SNP) molecular markers. The meiotic crossover events were investigated in a pollen grain population derived from “Eureka” lemon [*Citrus limon* (L.) Burm. f.] and the allelic SD was evaluated in a pollen grain population derived from a clementine × sweet orange hybrid (*C. clementina* × *C. sinensis*; hereafter referred to as “CSO” tangor). Data obtained from pollen grain population were compared to those obtained from genotyping of a segregating population recovered from a hand-made hybridization between diploid non apomictic selected tangor (*C. reticulata* × *C. sinensis*) as female parent pollinated with “CSO” tangor as male parent.

## Materials and Methods

### Plant Material

Plant material was sampled from the parental collection of our triploid breeding program (Navarro et al., 2015) carried out in the Instituto Valenciano de Investigaciones Agrarias (IVIA) located at Moncada (Valencia, Spain).

For crossover analysis, we isolated and whole-genome amplified pollen nuclei from the diploid “Eureka” lemon. Lemon resulted from an ancient direct interspecific hybridization between sour orange (*C. aurantium* L.) as female parent and citron (*C. medica* L.) as male parent (Nicolosi et al., 2000; Froelicher et al., 2011; Garcia-Lor et al., 2013b; Curk et al., 2016; Wu et al., 2018). Since sour orange and citron are genetically distant species, the identification of alleles from each parent is greatly facilitated (Wu et al., 2018).

On the other hand, for the SD analysis, we used a progeny of 86 diploid hybrids arising from a hand-made cross between diploid non apomictic selected tangor (*C. reticulata* × *C. sinensis*; hereafter referred to as “RTO” tangor) as female parent pollinated with “CSO” tangor as male parent, hereafter referred as “RTSO” population. This hybridization is of great interest in citrus breeding programs to combine desired attributes from both parents such as late maturing from “RTO” and anthocyanin content from “CSO.” Isolated and whole-genome amplified pollen nuclei from “CSO” tangor were genotyped to be compared with the diploid hybrids belonging to the “RTSO” population. Further information about recovery and ploidy level analysis of regenerated diploid plants can be found in Aleza et al. (2009b).

### DNA Extraction From Leaves

Genomic DNA was extracted from “RTO” and “CSO” tangors, “RTSO” diploid progeny, “Eureka” lemon, sour orange and citron, using a Plant DNeasy kit from Qiagen Inc. (Valencia, CA, United States) following the manufacturer’s protocol and measured using a spectrophotometer (NanoDrop 2000C, Thermo Fisher). The samples were diluted with sterile water (Sigma-Aldrich, Co., United Kingdom) at a concentration of 10 ng/μl and stored at −20°C until use.

### Fluorescence Activated Cell Sorting-Based Isolation of Single Pollen Nuclei and Whole Genome Amplification

For pollen grain recovery, between 40 and 50 flowers of the “CSO” tangor and “Eureka” lemon were collected in pre-anthesis during the spring of 2018. The anther’s were removed from the flowers with forceps and were dried in Petri dishes over silica gel in a desiccator at room temperature until the anthers opened after 1 or 2 days. Then, dehiscent anthers were selected under magnifying glass, discarding those that were not fully open, and they were sealed with parafilm and stored at −20°C until use. To isolate pollen nuclei we used the methodology described by Kron and Husband (2012) and validated in citrus by Garavello et al. (2019). Briefly, 4–5 dehisced anthers were vortexed in a small tube with 300 μl of nuclei isolation buffer in order to recover all pollen grains. Afterward, the suspension was filtered using the pre-filter and the bursting filter (CellTrics^®^ filters, Partec^®^). With the help of a plastic rod, collected pollen grains over the bursting filter were rubbed against the filter and washed with nuclei isolation buffer, twice. Subsequently, DAPI staining solution (1.5 μg/ml) was added to the suspension and incubated for 10 min. Stained suspension were run in a BD Influx (BD Biosciences, United States) and analyzed with BD FACS software.

We have followed the methodology described by Dreissig et al. (2015) to perform FACS-based purification of single nuclei and WGA. From the nuclei suspension, individualized nuclei were sorted into individual wells of a 384-microwell plate containing 2 μl lysis solution, which was composed of 0.5 μl lysis buffer, 0.5 μl ddH_2_O, and 1 μl sample buffer (Genomiphi V2, GE Healthcare). Illustra GenomiPhi V2 DNA Amplification Kit (GE Healthcare, United States) was used to WGA Prior to calculate the DNA concentration of WGA products, each sample was diluted with 500 μl of ddH_2_O, and subsequently was measured by fluorometric quantification (Qubit, Life Technologies).

Fifty-four pollen nuclei of “CSO” tangor and 44 pollen nuclei of “Eureka” lemon were sorted and whole genome amplified. Additionally, 10 single pollen nuclei of the analyzed genotypes were mixed in the same well to be used as a positive control against amplification errors (Dreissig et al., 2015).

### Genotyping Using Simple Sequence Repeat and Single Nucleotide Polymorphism Markers

The hybrids of “RTSO” progeny and their parents together with the amplified pollen nuclei of “CSO” tangor were genotyped using 30 molecular markers (23 SSRs and seven SNPs) heterozygous for “CSO” and with polymorphism with “RTO.” These markers are distributed across all linkage groups (LGs) of the Clementine genetic map (Ollitrault et al., 2012a). Isolated pollen nuclei of “Eureka” lemon were genotyped using seven SSRs and five SNPs heterozygous markers. These markers are distributed across LG1 of the Clementine genetic map (Ollitrault et al., 2012a). Detailed information about all markers used is given in Tables 1, 2.

**TABLE 1:** Information about molecular markers used for genotyping “CSO” tangor pollen nuclei, “RTSO” progeny and its parents, indicating accession number in Gene Bank or Phytozome, position in the reference clementine genetic map, noted alleles, and reference.

Locus	LG	Location (cM)	DC (cM)	Gene Bank/Phytozome accession	RTO noted alleles	CSO noted alleles	References
CIBE6147	1	14.39	46.27	ET085226	206-206	204-212	Ollitrault et al. (2010)
CIBE5720	1	58.69	1.97	ET082224	329-337	325-341	Ollitrault et al. (2010)
JK-TAA15	1	119.73	59.07	-	204-204	189-192	Kijas et al. (1997)
2P21022555	2	57.00	0.13	Ciclev10018135 m.g	GG	AG	Curk et al. (2015)
CX6F23	2	59.35	2.48	CF417259	162-168	155-162	Chen et al. (2008)
CIC3712-01	2	114.51	57.64	ET079481	CC	AC	Ollitrault et al. (2012b)
TAA41	2	160.74	103.87	–	154–160	148–154	Kijas et al. (1997)
MEST256	3	17.02	73.57	DY290355	225–225	210–225	García-Lor et al. (2012)
3P35931624	3	95.01	4.42	Ciclev10023979 m.g	GG	GA	Rouiss et al. (2017a)
JI-TC01	3	109.67	19.08	CK934237	333–342	333–352	In preparation
C4P5278891	4	18.44	2.3	–	GG	AG	Garavello et al. (2020)
CI03G05	4	75.07	58.93	FR677578	226–226	199–226	Cuenca et al. (2011)
CI03D12a	4	90.06	73.92	–	280–280	251–261	Aleza et al. (2011)
MEST15	5	16.21	6.91	FC912829	189–189	174–192	García-Lor et al. (2012)
MEST88	5	57.05	33.93	DY271576	104–112	112–118	García-Lor et al. (2012)
mCrCIR06A12	5	93.2	33.93	AM489742	95–95	95–102	Froelicher et al. (2008)
mCrCIR07E12	5	95.43	72.31	AM489750	118–118	118–124	Froelicher et al. (2008)
MEST191	6	10.79	4.59	DY283044	235–241	241–244	In preparation
MEST488	6	68.39	62.19	DY297637	126–126	126–140	García-Lor et al. (2012)
CIBE6256	6	84.49	78.29	ET085615	174–192	176–190	Ollitrault et al. (2010)
MEST107	7	8.89	87.54	DY274062	175–175	175–182	Cuenca et al. (2011)
MEST202	7	20.60	75.83	DY284147	169–172	157–169	In preparation
CIC3674-02	7	23.56	72.87	ET079224	GG	AG	Ollitrault et al. (2012b)
CI07C07	7	98.01	1.58	AJ567409	238–240	227–240	Froelicher et al. (2008)
mCrCIR07B05	8	33.17	21.04	AM489747	220–222	202–222	Froelicher et al. (2008)
CIC1208-01	8	57.78	3.57	ET070547	GG	AG	Ollitrault et al. (2012b)
mCrCIR02G02	8	59.15	4.94	FR677572	116–122	112–122	Cuenca et al. (2011)
CI02B07	9	0.01	52.15	AJ567403	162–170	160–162	Froelicher et al. (2008)
CIC5087-01	9	15.88	36.28	ET111514	TT	AT	Ollitrault et al. (2012b)
MEST308	9	50.41	1.75	DY296351	260-260	241-260	In preparation

*LG, Linkage group. Location and distance to the centromere (DC) derived from reference genetic map data (Ollitrault et al., 2012a) and location of centromere (Aleza et al., 2015).*

**TABLE 2:** Information about molecular markers used for genotyping “Eureka” lemon pollen nuclei, indicating accession number in Gene Bank or Phytozome, position in the reference clementine genetic map, noted alleles and reference.

Locus	LG	Location	DC	Gene Bank/Phytozome accession	Noted alleles	References
CIBE6126	1	2.68	57.98	ET084980	218–220	Ollitrault et al. (2010)
CIBE6147	1	14.39	46.27	ET085226	214–293	Ollitrault et al. (2010)
CiC4827-01	1	20.54	40.12	ET072918	AG	Ollitrault et al. (2012a)
1P3705568	1	32.48	28.18	Ciclev10010157 m.g	AG	Curk et al. (2015)
EMA-M30	1	46.03	14.63	JX630064	CT	Garcia-Lor et al. (2013a)
mCrCIR06B05	1	50.27	10.39	AM489744	187–199	Froelicher et al. (2008)
CIBE5720	1	58.15	2.51	ET082224	320–333	Ollitrault et al. (2010)
MEST539	1	61.82	1.16	DY294904	97–104	In preparation
MEST001	1	70.60	9.94	DY262452	171–187	Luro et al. (2008)
CIC5950-02	1	91.37	30.71	ET083949	GA	Ollitrault et al. (2012b)
TSC-C80	1	111.55	50.89	JX630084	TG	Garcia-Lor et al. (2013a)
MEST431	1	119.00	58.34	DY291553	331–348	García-Lor et al. (2012)

*LG, linkage group; DC, distance to the centromere [derived from reference genetic map data (Ollitrault et al., 2012a) and location of centromere (Aleza et al., 2015)].*

Polymerase chain reactions (PCRs) using SSR markers were performed using a Thermocycler rep gradient S (Eppendorf^®^) using the following protocol: reaction volume, 15 μl containing 0.5 μl of 1 U/μl of Taq DNA polymerase (Fermentas^®^), 3 μL of citrus template DNA (10 ng/μl), 1.5 μl of 2 mM welled (Sigma^®^) dye-labeled forward primer, 1.5 μl of 2 mM non-dye-labeled reverse primer, 0.2 mM of each dNTP, 1.5 μl of PCR reaction buffer 10×, and 0.45 μl of 50 mM MgCl_2_. The cycling program was set as follows: denaturation for 5 min at 94°C followed by 40 cycles of 30 s at 94°C, 30 s at 50°C or 55°C, 30 s at 72°C; and a final elongation step of 8 min at 72°C. Separation was carried out by capillary gel electrophoresis using a Genetic Analysis System 8000 (Beckman Coulter Inc.). The PCR products were initially denatured at 90°C for 2 min, injected at 2 kV for 30 s, and separated at 6 kV for 35 min. Alleles were sized based on a DNA size standard (400 bp). GenomeLab^TM^ v.10.0 (Beckman Coulter Inc.) genetic analysis software was used for data collection.

For SNP markers genotyping we used KASPar^TM^ technology by LGC Genomics^1^. Primers were directly designed by LGC Genomics from each SNP locus flanking sequence, considering approximately 50 nt on each side of the SNP. The KASPar genotyping system is a competitive allele-specific dual Förster resonance energy transfer (FRET)-based assay for SNP genotyping, and detailed explanation of specific conditions and reagents can be found in Cuppen (2007).

### Analysis of Segregation Distortion and Crossovers

The potential allelic SD was analyzed in the “RTSO” progeny as well as in the pollen nuclei population from “CSO” tangor using the Chi-square test (χ^2^), assuming an expected allelic segregation ratio of 1:1 (*p* < 0.05) for each analyzed marker.

The crossover events along the chromosome 1 were detected in “Eureka” pollen nuclei by identifying changes in alleles inherited from sour orange to citron, and vice versa. The number of crossovers was estimated for each arm of the chromosome 1 according to the following calculation:

$$C\,O\,r\,a\,t\,i\,o=\frac{n\,\left(\text{CO}\right)}{n\,\left(\text{M}\right)}$$

where *n* (CO) is the number of crossovers and *n* (M) is the total number of observations. The pairs of markers in the analyzed gametes that showed missing data points were omitted from the analysis.

### Analysis of Population Diversity

Population diversity organization between hybrids of the “RTSO” progeny at the male gamete level and the “CSO” pollen nuclei population was examined using DARwin6 software (Perrier and Jacquemound-Collet, 2018). Neighbor-joining analysis using the simple matching dissimilarity index (di-j) between pairs of markers (units) was performed:

$$d_{i-j}=1-\frac{1}{L}\,\sum_{l=1}^{L}\frac{m_{l}}{\pi }$$

where *d*_*i–j*_ is the dissimilarity between units *i* and *j*, *L* is the number of *loci*, *m*_*l*_ is the number of matching alleles for *locus l*, and *π* is the ploidy level, which is one in this case. From the dissimilarity matrix obtained, a Weighted neighbor-joining tree (Saitou and Nei, 1987) was computed.

## Results and Discussion

### Fluorescence Activated Cell Sorting of Pollen Nuclei, Whole Genome-Amplification, and Genotyping

In a previous work, we have demonstrated that FACS technique coupled with WGA is an adequate methodology for multi-locus SSR and SNP genotyping of citrus haploid pollen nuclei (Garavello et al., 2019). In the present work, a total of 44 and 54 haploid pollen nuclei from “Eureka” lemon and “CSO” tangor were analyzed, respectively.

**FIGURE 1:**
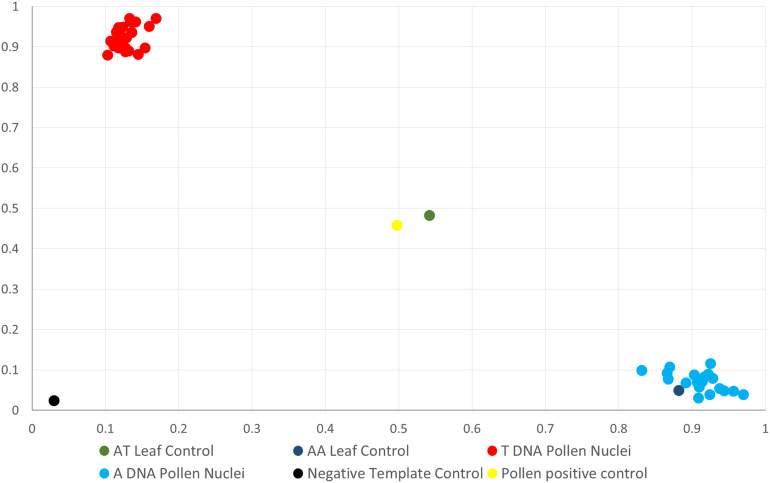
Representative dispersion diagram of PCR products obtained with CIC5087-01 SNP marker of haploid pollen grains nuclei amplified with WGA kit from the “CSO” diploid genotype.

“Clementine × sweet orange hybrid” tangor pollen nuclei were genotyped with the same set of molecular markers as the “RTSO” population (Table 1). For further analysis, we selected those gametes with a minimum of 65% positive amplifications (Figure 1) for the set of markers used. Thirty-four “Eureka” lemon (77.3%) and 48 “CSO” pollen nuclei (88.9%) showed at least 65% positive PCR reactions.

Out of 408 PCR reactions performed for all marker combinations in “Eureka” lemon (Table 2), 362 were positive (88.7%). On the other hand, the “CSO” tangor showed 1,393 positive PCR reactions (96.7%) of a total of 1,440.

The percentage of WGA positive PCR in “Eureka” lemon was slightly lower than that found by Dreissig et al. (2015) when analyzed barley pollen nuclei with SNP markers. However, this value was more similar for “CSO.” In the same way, these results are in agreement with what was found by Garavello et al. (2019), who conducted a classification and genotyping study of individual haploid citrus pollen nuclei.

**FIGURE 2:**
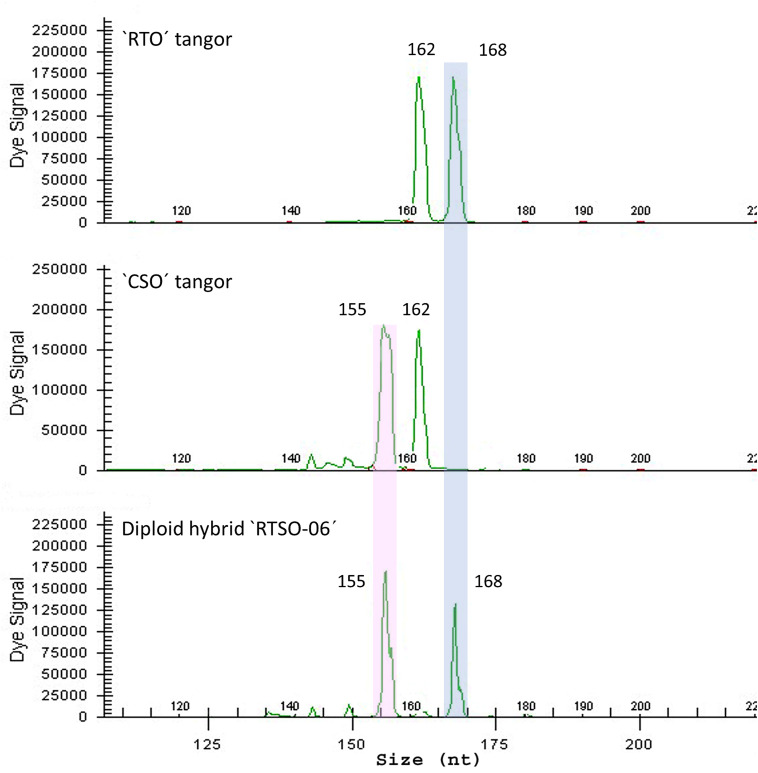
Electropherograms of a diploid hybrid “RTSO-06” recovered from hybridization between “RTO” diploid tangor as female parent and the “CSO” diploid tangor as male parent with CX06F23 SSR marker.

The genotyping of the 86 “RTSO” diploid hybrids and their parents, performed with the same set of markers used for “CSO” pollen nuclei (Table 1) allowed the unequivocal allelic differentiation between both parents and assessing the allelic contribution of the male parent to form each diploid hybrid (Figure 2).

### Analysis of Crossover Events in Haploid Pollen Nuclei of “Eureka” Lemon

We analyzed the number of crossovers for both arms on chromosome 1 (Table 3). The analysis of crossover rates revealed the presence of up to five crossovers events on one arm and four on the other arm. Comparatively, the average number of crossovers on chromosome 1 in the pollen nuclei population from “Eureka” lemon was 1.97.

**TABLE 3:** Number of observed crossover events on each arm of chromosome 1 based on analysis of haploid pollen nuclei from “Eureka” lemon using twelve SSR and SNP markers.

Number of crossovers		Arm 1					
		
		0	1	2	3	4	5
	**0**	5	3	1	0	0	0
	**1**	6	4	1	0	1	0
	**2**	6	2	1	0	0	1
**Arm 2**
	**3**	1	1	0	0	0	0
	**4**	1	0	0	0	0	0
	**5**	0	0	0	0	0	0

*Unbold numbers correspond with the number of crossovers on both arms in the haploid pollen nuclei population.*

Interestingly, we found five pollen nuclei (14.7%) with no crossover events between analyzed markers (Table 4), resulting with the same configuration as one of the lemon parents, i.e., citron or sour orange. Three pollen nuclei showed the same configuration as citron (samples Eur-P4, Eur-P9, and Eur-P19) while two pollen nuclei showed the same configuration as sour orange (samples Eur-P12 and Eur-P26) although it is possible that the crossovers in these pollen nuclei escaped to our detection, since in none of the five pollen nuclei all markers were amplified. The lack of amplification of these alleles may be attributed to the WGA kit performance at these specific loci since in the lemon leaf DNA PCR amplifications always happened (Table 4). In addition, other explanation could be related with the presence of null alleles in the parent species of the “Eureka” lemon. The remaining 29 pollen nuclei (85.3%) showed at least one recombination event between both genomes. However, our results indicate a tendency that were similar to those of Rouiss et al. (2017b) who, using six SSR markers detected up to four crossovers on one chromosome arm and three on the other arm when analyzing 27 unreduced gametes recovered from “Eureka Frost” lemon pollinated either with *C. ichangensis* or “Fortune” mandarin. Similarly, Aleza et al. (2015) and Cuenca et al. (2011) identified up to four crossovers on one chromosome arm in unreduced gametes of *C. clementina* and “Fortune” mandarin, respectively. Furthermore, Ollitrault et al. (2012a) identified up to two recombination break points on three LGs in the sweet orange gamete that originated *C. clementina*, and three crossover events on one LG in the *C. clementina* gamete that originated the haploid Clementine used by the International Citrus Genomic Consortium (ICGC) to establish the reference citrus haploid whole genome sequence (Aleza et al., 2009a).

**TABLE 4:** Multilocus configuration of the haploid pollen nuclei population from “Eureka” lemon analyzed with twelve SSR and SNP markers located on both arms of chromosome 1.

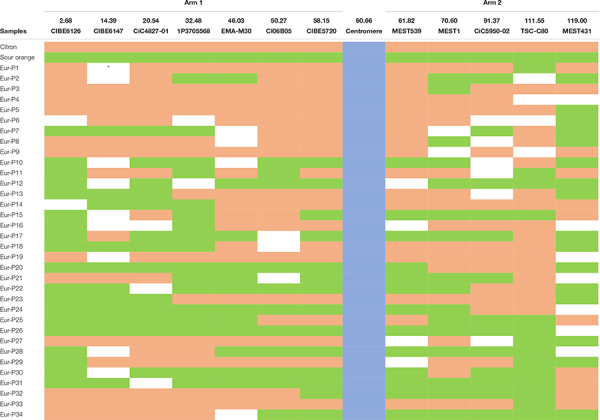

Light red indicates the presence of alleles inherited from citron and green indicates those from sour orange. *White boxes indicate no PCR product amplification with SSR or SNP markers.

In addition, in several organisms the frequency of meiotic crossover formation is determined genetically and also by the influence of environmental conditions. In plants, exposure to biotic and abiotic stresses can modify the overall rate of recombination (De Storme and Geelen, 2014). For example, a change in temperature from 22 to 30°C caused a reduction and alter distribution of meiotic crossover formation in barley (*Hordeum vulgare*) (Higgins et al., 2012), whereas a change in temperature from 18 to 28°C caused an increase of meiotic crossover formation in *Arabidopsis* (Francis et al., 2007). Similarly, in maize (*Zea mays*), both low temperature and water deficit significantly enhance the frequency of crossovers (De Storme and Geelen, 2014).

**FIGURE 3:**
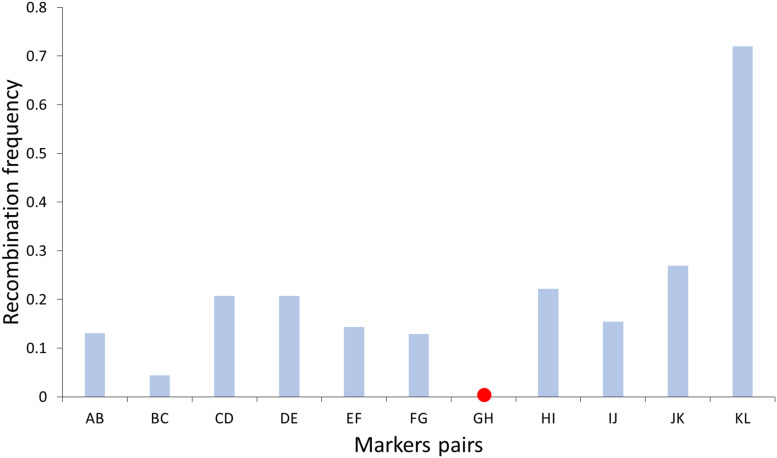
Recombination frequency along chromosome 1 determined by haploid pollen nuclei genotyping of “Eureka” lemon. Red point indicated relative position of centromere according to Aleza et al. (2015).

On the other hand, recombination frequency was determined along chromosome 1 by counting the number of crossovers at intervals of neighboring markers (Figure 3). The highest frequencies of recombination were found toward the distal region of the chromosome, while the lowest frequencies were found near the centromere, which is in agreement with what was observed in *C. clementina* where centromeric areas showed low recombination rates (<1.0 cM/Mb), although this reduction changed greatly between chromosomes (Aleza et al., 2015). Suppression of crossovers in centromeric and pericentromeric regions has been displayed in many plant species like wheat, barley, and tomato (Sherman and Stack, 1995; Dreissig et al., 2015; Darrier et al., 2017; Blary and Jenczewski, 2019).

This methodology is of great interest to study in citrus how environmental stress can alter the number and the distribution of crossover in haploid gametes and its implication in breeding programs based on sexual hybridization.

### Genotyping of “CSO” Haploid Pollen Nuclei Population and One Progeny of Diploid Hybrids Recovered With the Same Genotype as Male Parent

Conventional strategy to identify deviations from Mendelian segregation are based in genetic mapping populations from reciprocal and mixed crosses (Fishman and McIntosh, 2019). However, this conventional strategy can be improved by FACS, WGA, and genotyping of haploid pollen nuclei because with this new approach we might identify different mechanisms underlying SD that cannot be observed with the traditional way. To perform a comparative analysis between allele segregation in the “CSO” pollen nuclei population and the progeny recovered through hand-made pollination between “RTO” tangor as female parent and the “CSO” tangor as male parent (“RTSO” population), the same set of molecular markers was used distributed in the nine LGs of the Clementine genetic map (Ollitrault et al., 2012a). The preferential transmission of one allele over another in a statistically significant deviation from the expected Mendelian segregation ratio 1:1 is known as SD (Bodénès et al., 2016; Dai et al., 2017; Dreissig et al., 2017). Table 5 shows the number and all the Chi-square values of the “CSO” alleles in the pollen nuclei and the “RTSO” populations that were analyzed (Supplementary Tables 1, 2).

**TABLE 5:** Analysis of Mendelian allelic segregation (Chi-square test) for “CSO” tangor pollen nuclei population and “RTSO” progeny.

				CSO pollen nuclei population				RTSO progeny			
					
Locus	LG	Location	DC	A_1_	A_2_	Chi-square	*P* value	A_1_	A_2_	Chi-square	*P* value
CIBE6147	1	14.39	46.27	21	26	0.532	0.466	40	46	0.419	0.518
CIBE5720	1	58.69	1.97	27	21	0.750	0.387	41	45	0.186	0.666
TAA15	1	119.73	59.07	30	17	3.596	0.058	41	45	0.186	0.666
2P21022555	2	57.00	**0.13**	15	32	6.149	**0.013**	7	79	60.279	**0.000**
CX6F23	2	59.35	2.48	17	31	4.083	**0.043**	13	73	41.860	**0.000**
CIC3712-01	2	114.51	57.64	16	30	4.261	**0.039**	19	67	26.791	**0.000**
TAA41	2	160.74	103.87	21	27	0.750	0.387	52	34	3.767	0.052
MEST256	3	17.02	73.57	26	20	0.783	0.376	49	37	1.674	0.196
3P35931624	3	95.01	4.42	23	23	0.000	1.000	47	39	0.744	0.388
JI-TC01	3	109.67	19.08	23	22	0.022	0.882	42	44	0.047	0.829
C4P5278891	4	18.44	2.3	26	22	0.333	0.564	48	38	1.163	0.281
CI03G05	4	75.07	58.93	23	22	0.022	0.881	43	43	0.000	1.000
CI03D12a	4	90.06	73.92	25	21	0.348	0.555	41	45	0.186	0.666
MEST15	5	16.21	6.91	20	25	0.556	0.456	47	39	0.744	0.388
MEST88	5	57.05	33.93	31	16	4.787	**0.029**	40	46	0.419	0.518
mCrCIR06A12	5	93.20	70.08	24	23	0.021	0.884	38	48	1.163	0.280
CI07E12	5	95.43	72.31	25	21	0.348	0.555	42	44	0.047	0.829
MEST191	6	10.79	4.59	22	23	0.022	0.881	49	37	1.674	0.196
MEST488	6	68.39	62.19	25	20	0.556	0.456	50	36	2.279	0.131
CIBE6256	6	84.49	78.29	17	28	2.689	0.101	40	46	0.419	0.518
MEST107	7	8.89	87.54	24	23	0.021	0.884	4	82	70.744	**0.000**
MEST202	7	20.60	75.83	24	21	0.200	0.655	86	0	86.000	**0.000**
CIC3674-02	7	23.56	72.87	21	26	0.532	0.466	85	1	82.047	**0.000**
CI07C07	7	98.01	1.58	24	23	0.021	0.884	49	37	1.674	0.196
CI07B05	8	33.17	21.04	27	19	1.391	0.238	40	46	0.419	0.518
CIC1208-01	8	57.78	3.57	24	24	0.000	1.000	42	44	0.047	0.829
mCrCIR02G02	8	59.15	4.94	24	22	0.087	0.768	47	39	0.744	0.388
CI02B07	9	0.01	52.15	22	25	0.191	0.662	38	48	1.163	0.281
CIC5087-02	9	15.88	36.28	21	24	0.200	0.655	45	41	0.186	0.666
MEST308	9	50.41	1.75	22	25	0.191	0.662	47	39	0.744	0.388

*LG, linkage group; DC, distance to the centromere [derived from reference genetic map data (Ollitrault et al., 2012a) and location of centromere (Aleza et al., 2015)]; A_1_ and A_2_, number of individuals with that allelic configuration. Bold numbers indicate markers with SD.*

The markers displayed allelic SD in the two populations were analyzed. The rate of SD observed in “CSO” pollen nuclei (13.3%) was slightly lower than that recovered in “RTSO” population (20%). In the pollen nuclei population, SD was found on LGs 2 and 5, while the “RTSO” population showed SD on LGs 2 and 7. Globally, a total of seven analyzed markers showed SD. Among these markers, 2P21022555, CX6F23, and CIC3712-01, all located on LG 2, were synchronously distorted in the two populations. However, MEST88 marker, located on LG 5, shows SD for the pollen nuclei population, and not in the “RTSO” progeny. In addition, MEST107, MEST202, and CIC3674-02 markers, all located on LG 7, showed SD only in the “RTSO” population.

Segregation distortion can be originated by different processes that include non-random segregation of gametes during meiosis, alteration of viability or functionality of gametes after meiosis, and differential zygotic fitness (Fishman and McIntosh, 2019; Seymour et al., 2019). Synchronic SD visualized in the two gamete populations on LG 2 could be related with a gamete selection during pollen meiotic process. In other species like maize, monkeyflowers (*Mimulus* sp.), cotton (*Gossypium hirsutum*) it has been observed gametal factors that influence male gametes viability (Fishman and Saunders, 2008; Xu et al., 2013; Dai et al., 2017). For example, in monkey flowers Fishman and Saunders (2008) observed that the locus D strongly affects pollen viability and DD homozygotes suffered a 20% pollen viability reduction contributing to male fitness variation.

In addition, most loci with SD tend to cluster in segregation distortion regions (SDRs) as we have observed in both populations of “CSO” male gametes on LG 2. One explanation for SDRs could be that specific loci in the genome are conducted to viability differentiation (Luo and Xu, 2003; Li et al., 2011). The selection of an allele at the locus would result in nearby markers that deviate from the expected ratio, consistent with the theory of genetic hitchhiking (Dai et al., 2017).

Likewise, a strong SD has been observed on LG 7 only in “RTSO” progeny rather than “CSO” pollen nuclei population (Table 5, Supplementary Table 2). The gametophytic incompatibility system (GIS) was identified in citrus as one of the pollen–pistil interaction mechanisms that cause SD, defined as “the inability of a fertile hermaphrodite seed plant to produce zygotes after self-pollination” (Soost, 1965; de Nettancourt, 1977). Recently, Liang et al. (2019) have demonstrated that in citrus operate the S-RNase-based gametophytic self-incompatibility system that hold a S-RNase linked to about nine S-locus F-box genes. In this system, incompatibility reaction arises from the cytotoxic activity of S-RNase, meanwhile compatible pollen tubes avoid S-RNase cytotoxicity and grows into the style reaching the ovary (McClure et al., 2011). This system has been described in many genotypes including ancient and cultivated citrus species like mandarins and its hybrids (Liang et al., 2019). The GIS could be a factor for male gametic selection and this may lead to a complete exclusion of one allele for the concerned locus as we have noted in the three markers located on this LG with a very high distortion (4/82, 86/0, and 85/1 for MEST107, MEST202, and CIC3674-02 markers, respectively). In this context and taking into account the S-RNase-based GIS (Liang et al., 2019), “RTO” and “CSO” tangors have *C. sinensis* as the same ancestor and could share a S-RNase haplotype, whereby segregate as could be expected for the GSI system. These results could also suggest that in this genomic region could be located alleles related with the GIS in citrus. In fact, Liang et al. (2019) locate the S locus on LG 7 which is the same one we have observed SD. In previous results (data not shown) we could not recover any hybrid using “CSO” tangor as male parent in hand-made pollinations using clementines as female parent, suggesting incompatibility of this tangor with other genotypes. Nevertheless we cannot rule out zygotic or post-zygotic mechanisms involved in SD (Sweigart and Willis, 2012).

**FIGURE 4:**
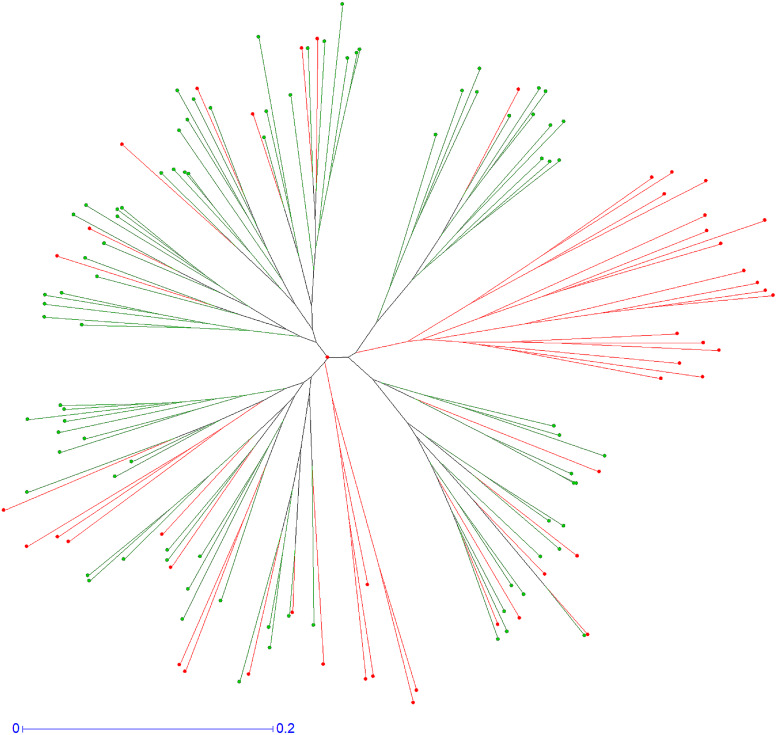
Neighbor-joining tree obtained from allelic data of “CSO” pollen nuclei population (red), and the male gametes from “RTSO” progeny (green).

On Figure 4 we display the genetic relationship of these two populations that was calculated by neighbor-joining analysis using the simple matching dissimilarity index, allowing the differentiation of gamete groups within each population and determining their genetic diversity. This figure shows a gamete cluster produced by “CSO” haploid pollen nuclei that were absent in the “RTSO” gamete population, allowing to conclude that in citrus some kind of selection occurs during the progamic phase, either in the pollen grain germination on the stigmatic surface, pollen tube growth into the style (GIS) or differences in the zygotic or post-zygotic viability, precluding the recovery of hybrids with specific allelic configurations.

Each gametes population showed different patterns of marker segregation, demonstrating that the genetic mechanism of SD has a specificity in each gametes population resulting from complex genetic system (Dai et al., 2017). These variations in the SD agree with different widely documented studies in plants, obtained through interspecific crosses for genetic mapping (Xu et al., 1997, 2013; Shirasawa et al., 2010; Bodénès et al., 2016; Dai et al., 2017; Fishman and McIntosh, 2019; Seymour et al., 2019), where large differences in SD values were found according to the direction of the crosses. The first distortion studies associated with sex were carried out in *Drosophila obscura* (Gershenson, 1928) because sexual dimorphism is common, and the sex ratio deviated a lot from 1:1 (Fishman and McIntosh, 2019; Seymour et al., 2019). Since then, several articles revealed a variety of meiotic and post-meiotic processes responsible for SD (Soltis et al., 1993; Fishman and Saunders, 2008; McDermott and Noor, 2010; Xu et al., 2013; Lindholm et al., 2016; Lambing et al., 2017). In citrus, Bernet et al. (2010) reported a higher SD in the male parent rather than in the female in a reciprocal hybridization between “Chandler” pummelo and “Fortune” mandarin. Subsequently, SD was evidenced by Ollitrault et al. (2012a) in clementine, pummelo and sweet orange when they were used either as female or male parents. From a breeding point of view, the presence SDRs implies that frequencies of relevant genes located there could be inherited at frequencies different from expected, affecting the efficiency of citrus breeding programs based on sexual hybridizations (Bernet et al., 2010).

In citrus, papers published until now about SD have been carried out mainly studying reciprocal crosses, as we have described before. In this work, we display for the first time a multi-locus genotyping study of the “CSO” haploid pollen gametes with and without the interference of the female parent. This approach allows us to identify mechanisms that could be related with specific genomic regions associated to non-random segregation of gametes during meiosis (as we have shown on LG 2) or with male and female gametic interactions, or zygotic mechanisms (LG 7). In addition, this methodology may have an important advantage for the achievement of sequencing projects. High heterozygosity is a general characteristic of Citrus species, making it difficult to assemble large genome sequences. For this reason, the ICGC decided to establish a reference whole citrus genome sequence from a clementine homozygous plant recovered by *in situ* gynogenesis induced by irradiated pollen Aleza et al. (2009a). Therefore, obtaining haplotypes from FACS coupled with WGA of haploid pollen nuclei would allow the sequencing projects to be approached more easily, from any genotype regardless its heterozygosity, and without the need to recover haploid plants using *in vitro* techniques, which in many cases is a very difficult task with very low efficiency.

## Conclusion

Fluorescence activated cell sorting coupled with WGA allows the analysis of individual nuclei with a large number of molecular markers, offering the opportunity to efficiently determine the frequency of crossovers and the SD in haploid pollen nuclei without the need to generate segregating populations.

Until now, no study was conducted regarding the recombination in citrus pollen nuclei. The analysis of the “Eureka” lemon pollen nuclei allowed the identification of recombination points through the use of SSR and SNP markers, showing a greater number of crossovers in centromere distal regions of chromosome 1. In addition, SD has been observed either on LG 2 of both populations and only on LG 7 we have observed that the SD in the pollen of “CSO” tangor and the plants of the population differ in frequency or position. Potential male gametic selection mechanisms were distinguished in pollen grains, while in the population, mechanisms of gametophytic selection and/or zygotic selection were observed. The methodology presented here represents a very useful tool to facilitate research focused on the reproductive biology of citrus and study the mechanisms that affect crossovers and SD.

## Data Availability Statement

All datasets generated for this study are included in the article/Supplementary Material.

## Author Contributions

MG and PA conceived the study and were in charge of the direction and planning. JF isolated pollen nuclei and conducted flow-sorting. SD performed whole genome amplification from single pollen nuclei. MG, JC, and PA genotyped pollen nuclei, diploid hybrids, analyzed the data, and took the lead writing the manuscript with input and review of LN, SD, JF, and AH. All authors read and approved the final version of this manuscript.

## Conflict of Interest

The authors declare that the research was conducted in the absence of any commercial or financial relationships that could be construed as a potential conflict of interest.

## Footnotes

1 http://www.lgcgenomics.com
